# AURKB Enhances Chromosomal Remodeling of Telomeric Genes and Accelerates Tumorigenesis of Uveal Melanoma

**DOI:** 10.1167/iovs.64.4.23

**Published:** 2023-04-20

**Authors:** Huixue Wang, Hui Pan, Xiaolin Huang

**Affiliations:** 1 Department of Ophthalmology, Ninth People's Hospital, Shanghai Jiao Tong University School of Medicine, Shanghai, China; 2 Shanghai Key Laboratory of Orbital Diseases and Ocular Oncology, Shanghai, China

**Keywords:** uveal melanoma, AURKB, histone modification, telomere, target therapy

## Abstract

**Purpose:**

Uveal melanoma (UM) is the most common intraocular malignancy in adults developing liver metastases, threatening a patient's life. Current therapeutics failed to significantly improve the survival of patients with UM. Thus, the discovery of potent drugs is imminent.

**Methods:**

Integrated bioinformatic analysis of The Cancer Genome Atlas and immunohistochemistry staining of patients' tissues revealed the oncogenic role of aurora kinase B (AURKB) in UM. Drug sensitivity assays and an orthotopic intraocular animal model were used to test the efficacy of AURKB inhibitors. RNA sequencing and immunoblotting were performed to identify the downstream effector. A chromatin immunoprecipitation assay was conducted to elucidate AURKB's transcriptional regulation on the target gene.

**Results:**

AURKB was found overexpressed in patients with UM, resulting in a poor prognosis. Luckily, the AURKB-specific inhibitor, hesperadin, achieved prominent pharmacological efficiency in UM in vitro and in vivo. Mechanically, hesperadin compromised phosphorylation of histone H3 at serine 10 (H3S10ph) at the promoter of telomerase reverse transcriptase, accompanied by methylation of histone H3 at lysine 9. This methylated status of the promoter region forced chromatin condensation and consequently halted the transcription of telomerase reverse transcriptase.

**Conclusions:**

Collectively, our data demonstrated that AURKB inhibitors decelerated UM tumorigenesis by epigenetically silencing the expression of oncogenic telomerase reverse transcriptase, indicating AURKB as a potential therapeutic target in UM.

Uveal melanoma (UM) is adults' most common primary intraocular malignancy, severely impairing visual function and even a patient's life.^1^ Approximately one-half of patients with UM end up with distant metastases, and suffer death within 1 year.^1^^–^^4^ Thus, the early detection and treatment of small lesions are crucial to local control and visual preservation, metastasis prevention, and survival improvement.

The aurora kinase family contains three members: aurora kinase A/B/C, which possess a conserved catalytic domain but perform distinct functions.^5^ As a serine/threonine kinase, aurora kinase B (AURKB) is well-known for its physiological role in mitotic progression, including spindle assembly, chromosome alignment, and cytokinesis, through highly organized spatiotemporal phosphorylation of interacting proteins at the centromere region.^5^ Furthermore, AURKB loss leads to abnormal mitosis and chromosome missegregation, resulting in genetic instability.^6^ Besides the pivotal role in mitosis, AURKB can be activated by the ATR-CHK1 pathway in response to DNA replication errors.^7^ In addition, activated AURKB in the midbody triggers the abscission checkpoint, avoiding micronuclei formation.^8^

Recently, AURKB amplification or overexpression has been reported frequently in various malignancies, suggesting a higher tumor stage and a poor prognosis. In addition, AURKB, in cooperation with mutant TP53, drove the malignant transformation of Chinese hamster embryo cells in vivo.^9^ Moreover, certain chemotherapy-resistant tumors showed elevated expression of AURKB or growth arrest to AURKB inhibitors.^10^ Because the aberrant expression of AURKB was observed frequently in malignancies, a variety of small molecule inhibitors targeting AURKB have been developed and are in different phases of clinical trials.^11^ Hesperadin, primarily used as an adenosine triphosphate–competitive indolinone inhibitor of AURKB, interfered the active conformation of the T loop marked by phosphorylation on Thr232.^12^^,^^13^ Apart from AURKB, hesperadin inhibited other kinase activity, including CaMKII-δ, MST4, MEKK2, AMPK, Lck, MKK1, MAPKAP-K1, CHK1, and PHK.^14^^–^^16^ In addition, hesperadin suppressed pancreatic cancer through a novel ATF4/GADD45A axis mediated by excess mitochondrial–endoplasmic reticulum stress.^17^ Several studies have demonstrated AURKB's role in activating the PI3K/AKT/nuclear factor-κB pathway to promote survival and inhibit apoptosis.^18^ However, the role of AURKB in UM and the underlying mechanism remain elusive.

In the present study, an integrated analysis of The Cancer Genome Atlas (TCGA) suggested AURKB's role in accelerating oncogenesis and metastasis of UM, further confirmed by a series of in vitro and in vivo experiments. RNA sequencing of UM cells treated with AURKB inhibitor indicated that AURKB might manipulate telomere organization. Further, a chromatin immunoprecipitation (ChIP) assay demonstrated that AURKB achieved this sophisticated regulation by modulating histone's epigenetic status, creating an open chromatin structure to facilitate transcription of telomerase reverse transcriptase (TERT). Therefore, our findings characterize a profound transcription-activated network that links tumorigenesis and telomere homeostasis through AURKB-mediated chromatin remodeling and lay the theoretical foundation for the potential clinical application of AURKB inhibitors in UM.

## Methods

### Reagents

Danusertib, hesperadin, TAK-901, and Aurora A inhibitor I were purchased from MedChemExpress Company (Monmouth Junction, NJ, USA). Primary antibodies included rabbit anti-phospho-AURKB^T232^ (Cell Signaling Technology, Danvers, MA, USA; 2914T), mouse anti-AURKB (Abcam [Cambridge, UK], 3609), rabbit anti-H3 (Abcam, ab1791), rabbit anti-H3S10ph (Abcam, ab5176), TERT (Abcam, ab230527), histone H3 at lysine 9 (H3K9me)2/3 (CST, 13969), and GAPDH (Bioworld Technology, Bloomington, MN, USA; MB001). Secondary antibodies included HRP-linked rabbit IgG (Cell Signaling Technology, 7074) and HRP-linked mouse IgG (Cell Signaling Technology, 7076).

### Cell Culture

Cell lines were grown in Roswell Park Memorial Institute 1640 medium containing 10% fetal bovine serum and 1% antibiotics (penicillin, 100 U/mL; streptomycin, 100 µg/mL). These cells were kept in a humidified environment at 37°C with 5% CO_2_ and 100% humidity. The culture medium was changed every 3 days.

### Drug Sensitivity Half Maximal Inhibitory Concentration Data

Drug sensitivity was tested in UM cells using CCK8 assay. Briefly, cells were seeded in 96-well plates at a density of 5000 cells/well and then treated with different concentrations of drugs in triplicate. After 72 hours of incubation, cell viability was quantified using CCK8 assay, and the half maximal inhibitory concentration was estimated using GraphPad Software 8.0.1.

### Flow Cytometry

Cells were collected and fixed in 70% ethanol, treated with 300 µg/mL RNase A, and their nuclei were stained with 10 µg/mL PI. A FACS cytometer detected the stained nuclei. The data were analyzed by ModFit software. The apoptosis assay was performed using a FITC Annexin V Apoptosis Detection Kit (BD Bioscience, Franklin Lakes, NJ, USA). Cells were washed twice with cold PBS and then resuspended in 1 × binding buffer at a concentration of 1 × 10^6^ cells/mL, and 5 µL of Annexin V-FITC conjugate and 10 µL of PI solution were added to each 100 µL cell suspension. The cells were gently vortexed and incubated for 15 minutes at room temperature in the dark. Next, 400 µL of 1× binding buffer was added to each tube. Flow cytometry was used to analyze the cells within 1 hour, and the concrete apoptosis rate was calculated using FlowJo software.

### RNA Sequencing and Bioinformatic Analysis

A total of 92.1 cells were treated with DMSO or 0.1 µM hesperidin for 24 hours. Total RNA was extracted, and the mRNA expression profile was analyzed using RNA-sequencing. A gene set enrichment analysis was performed using the online tool WebGestalt (http://www.webgestalt.org/).^19^

### Database Analysis

The GEPIA (http://gepia.cancer-pku.cn/) database provides UM survival information and correlation indexes. Kaplan–Meier survival analyses of AURKB and TERT were analyzed, and the cutoff value was set according to the median value of the normalized expression. In the TCGA, patients with UM are divided into two groups according to the Z-score of AURKB. The vital status, metastatic event, SCNA cluster, BAP1 mutation, and transcriptome of these two groups were then compared.

### Immunohistochemistry (IHC)

Briefly, the formalin-fixed paraffin-embedded tissue sections were dewaxed and rehydrated, followed by heat-induced antigen retrieval at a pH of 9.5, then the primary antibodies AURKB and H3S10ph were added. A few sections were incubated with 5% BSA as negative controls. Paraffin-embedded human colorectal cancer tissues were used as positive controls owing to high AURKB expression (www.proteinatlas.org). Next, the tissue sections were incubated for 8 hours at room temperature. Next, the tissue sections were incubated with goat anti-mouse IgG Alexa Fluor 488 (Abcam, ab150113) and goat anti-rabbit IgG Alexa Fluor 647 (Abcam, ab150079) for 90 minutes in the blocking buffer. Next, the tissue sections were stained with Hoechst for 30 minutes in a wash buffer. Then, the tissue sections were immersed in ethanol until the desired staining was reached. Finally, the tissue sections were coverslipped with appropriate water-based mounting media. The relative quantification of immunofluorescence tissue staining was achieved by Image J Software.^20^^,^^21^ Written informed consents were provided by all participants involved in this study.

### Immunoblotting

Proteins were extracted via protein lysis buffer. The supernatants were collected after centrifuging the lysates at 12,000×*g* for 5 minutes at 4°C. The protein concentration was assessed using the BCA Protein Assay Kit (Thermo Fisher Scientific, Waltham, MA, USA). Cell lysates containing 30 µg protein were separated on a 4-20% sodium dodecyl sulfate-polyacrylamide gel electrophoresis gel (Solarbio, Beijing, China) and then transferred onto polyvinylidene difluoride membranes (Millipore, Burlington, MA, USA) using a Trans-Blot Turbo. Membranes were then blocked in a solution of Tris-buffered saline containing 0.05% Tween-20 and 5% skimmed milk for 60 minutes at room temperature. Primary antibodies were incubated overnight at 4°C. Secondary antibodies conjugated with HRP were incubated with the membranes for 2 hours at room temperature. Membranes were finally developed using an enhanced chemiluminescence substrate.

### ChIP

The ChIP assay was performed using the EZ-Magna ChIP A/G Kit (Millipore) according to the manufacturer's instructions. Cells were fixed and centrifuged, and the pellets were resuspended in ChIP lysis buffer. After sonication, the sheared DNA complex of nuclear lysates was collected, and antibodies such as H3S10ph, H3K9me2/3, RNA polymerase II, and IgG were added. The mixture was incubated overnight, and magnetic beads were added to pull down the DNA–protein–antibody complexes. After elution, the samples were ready for real-time PCR analysis. The sequences of ChIP primers are presented here: forward, 5ʹ-TCCCCTTCACGTCCGGCA-3ʹ; reverse, 5ʹ-AGCGGAGAGAGGTCGAATCG-3ʹ.

### The Relative Telomere Length by Quantitative RT-PCR

Quantitative PCR was conducted in triplicate, and the reactions included 4 µL of genomic DNA (80 ng), 0.1 µL of telomere primer (10 µM) (forward: 5ʹ-CGGTTTGTTTGGGTTTGGGTTTGGGTTTGGGTTTGGGTT-3ʹ; reverse: (GGCTTGCCTTACCCTTACCCTTACCCTTACCCTTACCCT-3ʹ), 0.1 µL of YH-1 forwards primer (10 µM) (5ʹ-CGCACAGAGTAGTAAGGAAAGTGAAGTAGGCCGGGC-3ʹ), 1 µL of YH-1 reverse primer (10 µM) (5ʹ-GTGCTGGGATTACAGGCGTGAG-3ʹ), 1 µL of uniprimer2 (5ʹ-VIC-ATGGACAGTGAGATCTGTCCAT-BHQ1CGCACAGAGTAGTAAG-3ʹ), and 10 µL NovoStart SYBR quantitative PCR SuperMix, in a final reaction volume of 20 µL. The telomere amplifications were detected using SYBR green dye. All PCRs were carried out on a 7500 Real-Time PCR System. More information was found in Xiao's work.^22^

### Orthotopic Intraocular Animal Model

A total of 92.1 cells (2.5 × 10^5^ in 2.5 µL per injection) were inoculated into the subretinal space of the right eyes of 8-week-old female nude mice on day 0. To prepare a needle track, a 30G needle was introduced behind the limbus to access the choroid, and the cell suspension was inoculated using a Hamilton microliter syringe through the needle track. On days 7, 14, 21, and 28, and the experimental group received 2.5 µL hesperadin (10 mM) by intravitreal injection, the control group received an equal volume of saline. On day 35, the mice were humanely killed for eye enucleation. The Animal Care and Use Committee at Shanghai Jiao Tong University School of Medicine approved the study.

### Small Interfering RNA (siRNA) and Transfection

siRNA duplexes for knockdown of AURKB were synthesized by GenePharma (Suzhou, China). Cells were transfected with the siRNAs using Lipofectamine 2000 reagent (Invitrogen, Waltham, MA, USA) according to the manufacturer's instructions. The sequences of si-AURKB-1 and -2 are UUGAUGACUUUGAGAUUGG and GGAGGAGGAUCUACUUGAUU.

### Lentivirus Package

The 293 T cells were maintained in complete DMEM culture medium at a concentration of 6,000,000 cells per plate, and transfected using Lipofectamine 2000 reagent with 3 mg of pLVX-TERT, 3 mg of pMD2.D, and 6 mg of PsPax. The medium was replaced with 5 mL fresh medium after 6 hours' incubation with 293T cells. The supernatants were collected at 48 hours and 72 hours after transfection and then mixed and filtered through a 0.45 µm cellulose acetate filter (Sartorius, Göttingen, Germany). The viral supernatants were further concentrated with Amicon Ultra-15 Centrifugal Filter Units (Millipore) at 4°C and spun at 5000 rpm for 30 minutes. The colonies were selected for subsequent culture after incubation with 1 µg/mL puromycin for 2 weeks.

### Statistical Analyses

Statistical analysis was conducted using GraphPad Software 8.0.1. Data were presented as the mean ± SEM or SD. In addition, the significant difference between the treated and the control was tested by the Student *t*-test. A *P* value of less than 0.05 was considered statistically significant.

## Results

### AURKB Indicated Poor Survival of Patients With UM

**Figure 1.**
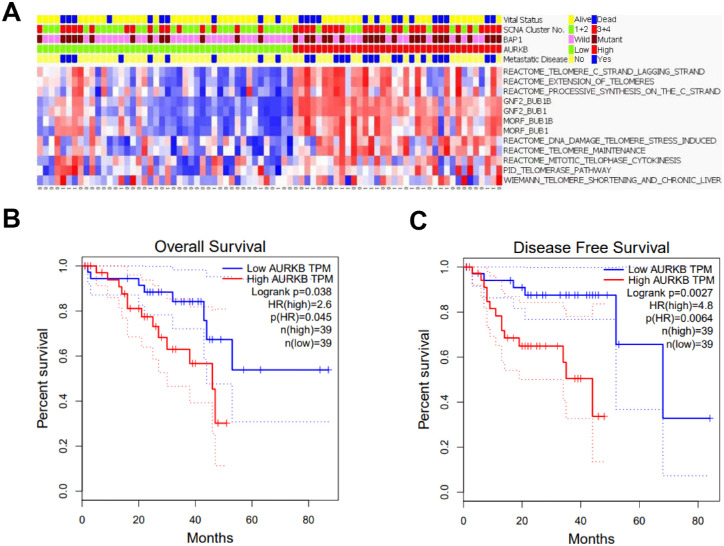
AURKB indicated poor survival of patients with UM. **(A)** Integrated analysis of TCGA demonstrated that high hierarchical SCNA cluster and BAP1 mutation were more frequent in patients with higher AURKB expression, who were susceptible to metastasis and death. Kyoto Encyclopedia of Genes and Genomes (KEGG) pathway analysis suggested that the telomere program signature was enriched for the high-AURKB subgroup. **(B**, **C)** Kaplan–Meier survival analysis according to GEPIA suggested that AURKB expression was negatively correlated with overall survival (**B**) and disease-free survival **(C)** of patients with UM.

To explore the possible role of AURKB in UM, we first classified the UM samples in TCGA into two groups according to the mRNA expression level of AURKB, and found that high-AURKB group possessed higher rate of death and metastasis (Fig. 1A). To further confirm the role of AURKB in UM prognosis, we queried the GEPIA database.^23^ AURKB expression was negatively correlated with overall survival and disease-free survival of patients with UM (Figs. 1B, 1C). Somatic copy number alterations (SCNA) were recurrent in UM, including losses in 1p, 6q, 8p, and 16q; gains in 6p and 8q; and M3.^24^ Unsupervised SCNA clustering defined four subtypes, with SCNA clusters 3 and 4 harboring more aneuploid events associated with metastasis.^24^ Integrated analysis of TCGA indicated that SCNA clusters 3 and 4 were more frequent in the high AURKB group (Fig. 1A). Additionally, inactivating BAP1 mutation was also considered as an important marker of UM metastasis,^24^ which was more frequent in specimens with higher AURKB expression (Fig. 1A). We finally performed Kyoto Encyclopedia of Genes and Genomes pathway analysis in TCGA and revealed that genes compromising telomere program signature, widely accepted for its role in cancer initiation and metastasis, were highly enriched in UM samples with higher AURKB level (Fig. 1A). Altogether, these data indicate that AURKB could play a pivotal role in modulating UM tumorigenesis and metastasis.

### UM Cells Were Sensitive to AURKB Inhibition

**Figure 2.**
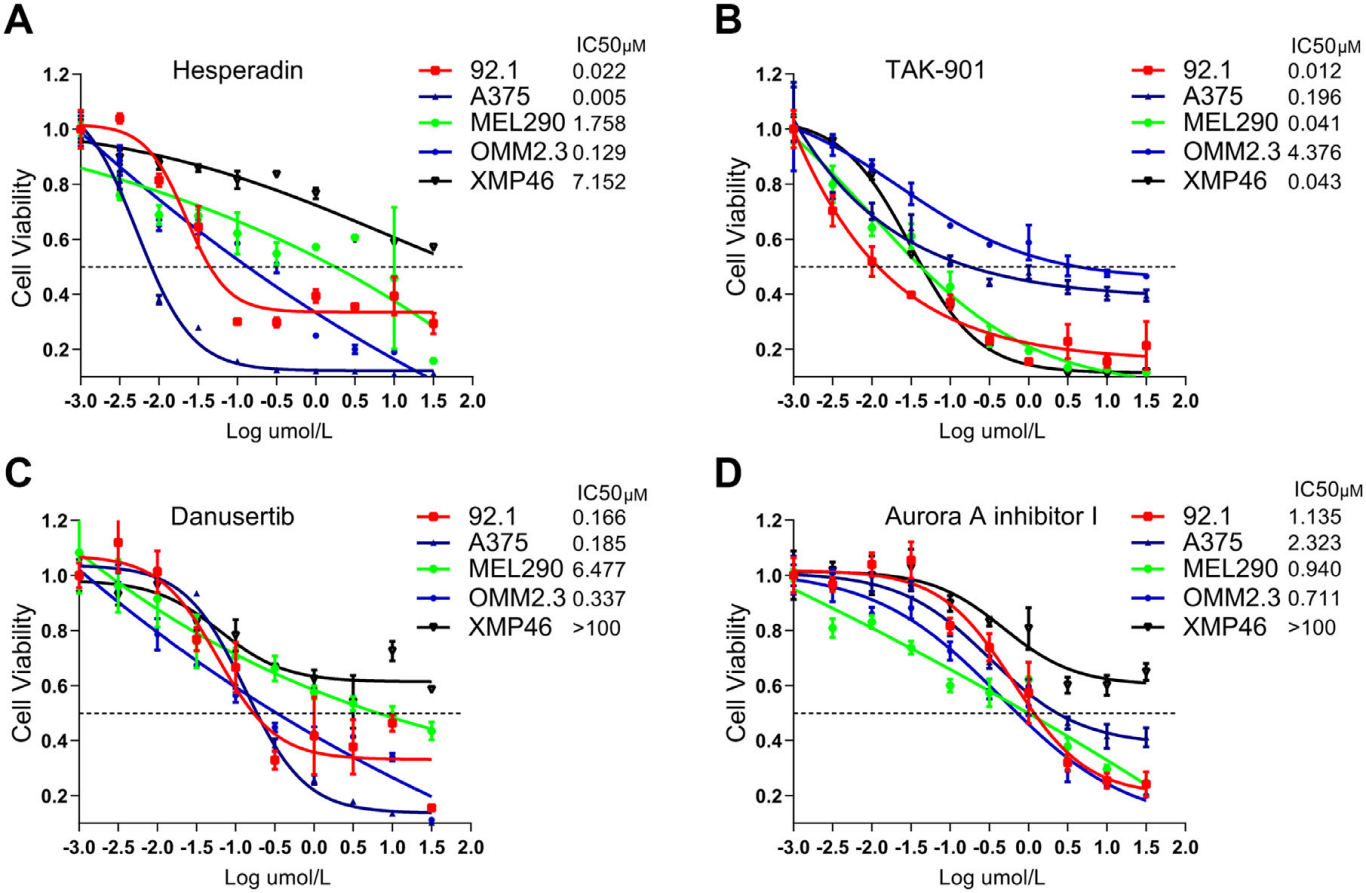
UM cells were sensitive to AURKB inhibitors. Four aurora kinase inhibitors, including AURKB-specific inhibitor hesperadin **(A)**, dual-target inhibitor targeting AURKA/B TAK-901 **(B)**, pan-aurora kinase inhibitor danusertib **(C)**, and AURKA-specific inhibitor Aurora A inhibitor I **(D)** were examined for their antitumor efficacy on four UM cell lines (92.1, MEL290, OMM2.3, and XMP46) and one cutaneous melanoma cell line A375. The half maximal inhibitory concentration (IC50) values were calculated using GraphPad Software 8.0.1.

We performed the drug sensitivity assay of AURKB inhibitors on four UM cell lines (92.1, MEL290, OMM2.3, and XMP46) and one cutaneous melanoma cell line (A375).^18^ TAK-901, a dual target inhibitor targeting AURKA and AURKB, had the strongest antitumor activity (Fig. 2B). AURKB-specific inhibitor hesperadin also showed a low half maximal inhibitory concentration in most UM cell lines ranging from 5 nM to 7 µM (Fig. 2A). Danusertib, a potent pan-aurora kinase inhibitor with the strongest activity against AURKA, was less efficient in inhibiting UM cells than TAK-901 and hesperadin (Fig. 2C). The AURKA-specific inhibitor, Aurora A inhibitor I, possessed the lowest tumoricidal activity (Fig. 2D). These results demonstrate that UM cells are sensitive to inhibitors specifically targeting AURKB rather than AURKA/C. To further explain the varied therapeutic response in different cells, the baseline protein level of AURKB was detected (Supplementary Fig. S1A). As expected, the sensitivity of melanoma cells to hesperadin was positively correlated with AURKB expression. In addition, we detected the AURKB's active form in UM cells exposed to four inhibitors at the same concentration series (Supplementary Fig. 1B). Hesperadin, TAK-901, and danusertib decreased the protein level of phospho-AURKB^T232^ to varying degrees, basically in consistent with drug sensitivity. However, no significant change of phospho-AURKB^T232^ was detected in cells treated with Aurora A inhibitor I, although UM cells were moderately sensitive to this inhibitor. To avoid off-target effects, siRNAs were used to specifically knock down AURKB (Supplementary Fig. S1C). A CCK8 assay showed that AURKB knockdown severely inhibited the proliferation of UM cells (Supplementary Fig. S1D), which proved AURKB's oncogenic role in UM. Collectively, both genetic and pharmacological approaches demonstrated AURKB's central role in UM carcinogenesis.

### Hesperadin Induced UM Cell Cycle Arrest and Apoptosis

**Figure 3.**
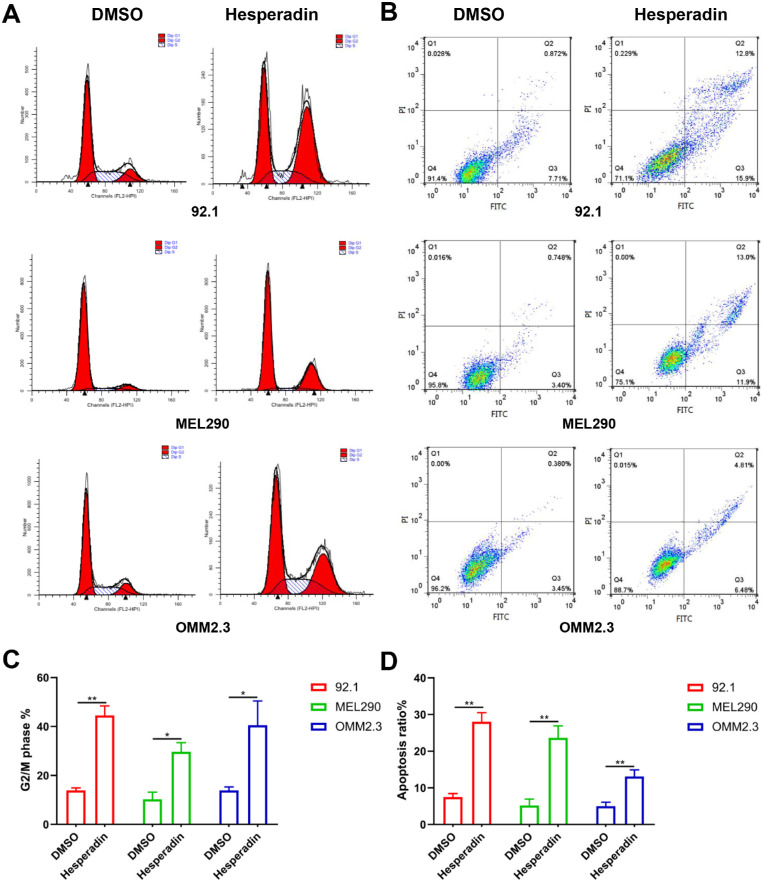
Hesperadin-induced UM cell cycle arrest and apoptosis. **(A)** 92.1, MEL290, and OMM2.3 cells were treated with 0 and 0.3 µM hesperadin. After 24 hours, the cells were collected and stained with PI. Then, the cell cycle distribution was measured using flow cytometry. **(B)** Cells were also stained with Annexin V-FITC/PI and measured using flow cytometry. The living cells were located in the lower left quadrant. **(C)** Quantification of cells in the G2/M phase. **(D)** Quantification of early and late apoptotic cells located in the lower and upper right quadrants. Statistical analysis was conducted using GraphPad Software 8.0.1. Data were presented as the mean ± SD. In addition, the significant difference between the treated and the control was tested by the Student *t*-test. *P < 0.05; **P < 0.01.

To further explore the role of AURKB in UM, we chose the AURKB-specific inhibitor hesperadin to study the impact on the cell cycle and apoptosis. After treatment with hesperadin in 92.1, MEL290, and OMM2.3 cells, the percentages of G2/M phase cells significantly increased. Correspondingly, the percentage of cells in G1 phase decreased (Figs. 3A, 3C). Annexin V-FITC/PI staining showed that hesperadin could induce 92.1, MEL290, and OMM2.3 cell apoptosis, with apoptosis rates of approximately 28.7, 24.9, and 11.29 (Figs. 3B, 3D). These results demonstrate that hesperadin could inhibit the propagation of UM cells by inducing cell cycle arrest and apoptosis.

### Hesperadin Regulated Transcriptomic Signatures Related to DNA Replication and Mitosis

**Figure 4.**
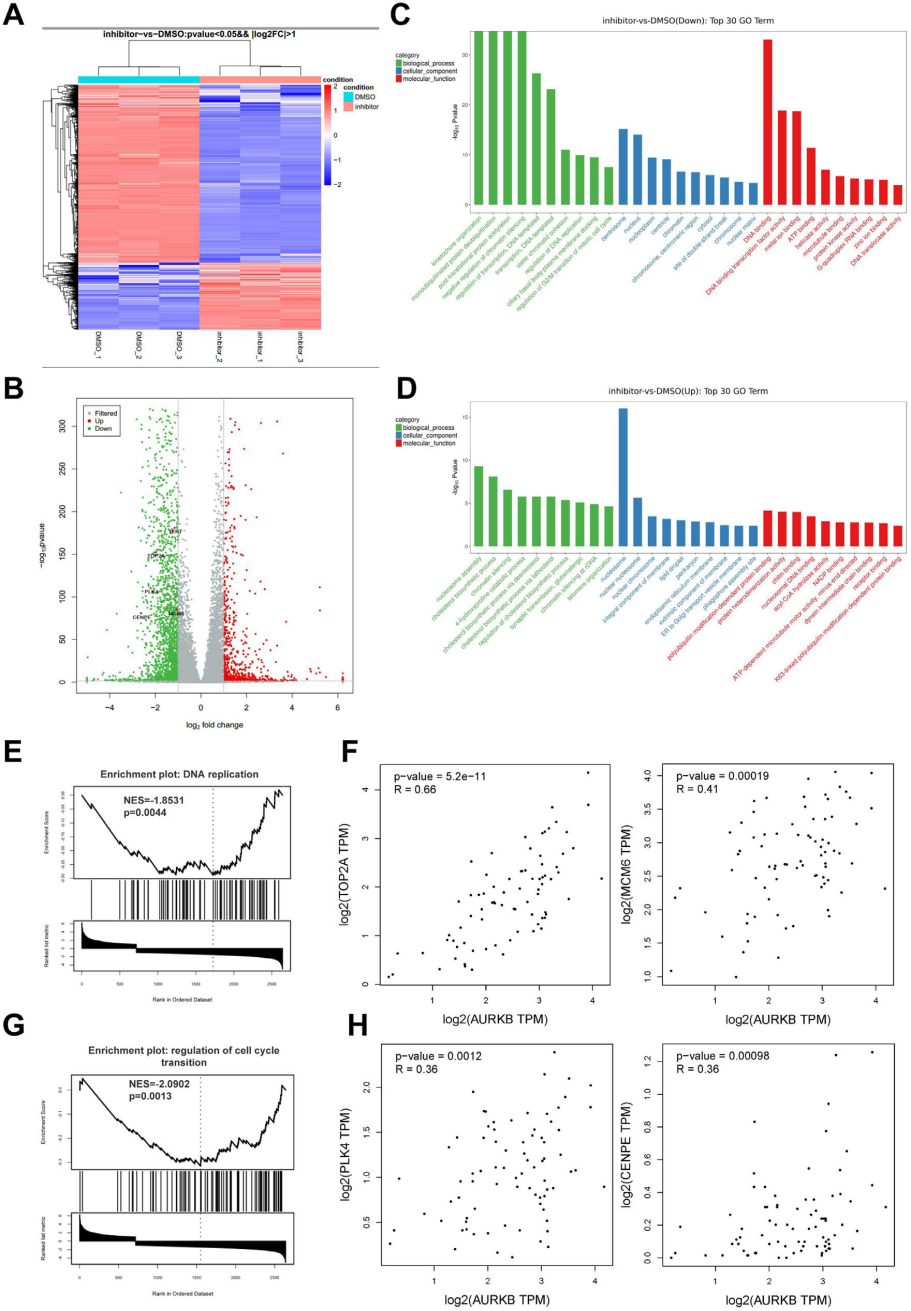
RNA-sequencing revealed that hesperadin changed the transcriptomics of UM cells. **(A)** Heat map of RNA-sequencing data performed on 92.1 cells treated with DMSO or 0.1 µM hesperadin for 24 hours. **(B)** Volcano plot of RNA sequencing data presented 1967 downregulated and 742 upregulated DEGs with a *P* value of less than 0.05 and |log2FC| > 1. **(C**, **D)** Gene ontology (GO) pathway functional enrichment analyses were performed to interpret the biological process, cellular components, and molecular function of transcripts and relative transcript abundance for downregulated **(C)** and upregulated **(D)** DEGs. **(E)** Gene set enrichment analysis (GSEA) analysis of DEGs hesperadin's effect on DNA replication. **(F)** GEPIA revealed that TOP2A and MCM6 were positively correlated with AURKB. **(G)** GSEA analysis of DEGs hesperadin's effect on cell cycle transition. **(H)** GEPIA revealed that PLK4 and CENPE were positively correlated with AURKB.

To evaluate transcriptome changes of UM cells in response to hesperadin, we treated 92.1 with 0.1 µM hesperadin or DMSO for 24 hours and then performed RNA sequencing on both groups in triplicate. Heat map of all differentially expressed genes (DEGs) with a *P* value of less than 0.05 and |log_2_FC| of greater than 1 was shown in Figure 4A. A total of 1967 downregulated and 742 upregulated DEGs were identified (Fig. 4B). Gene ontology pathway functional enrichment analyses were performed to interpret transcripts' biological process, cellular components, and molecular function, and relative transcript abundance. Downregulated genes owing to hesperadin exposure were enriched for kinetochore organization, sister chromatid cohesion, regulation of DNA replication, and cell cycle transition in terms of biological process, consistent with AURKB's role in mitosis regulation (Fig. 4C). Upregulated transcripts were primarily associated with nucleosome assembly and cholesterol metabolism (Fig. 4D).

Next, we further performed gene set enrichment analysis of DEGs. Gene ontology analysis in the biological process revealed that DNA replication and cell cycle progression gene sets were downregulated due to hesparadin treatment (Figs. 4E, 4G). Two well-known DNA replication regulation genes, TOP2A and MCM6, were inhibited transcriptionally, both of which had a high correlation intensity with AURKB in terms of transcript abundance in GEPIA database (Fig. 4F). Moving on to cell cycle transition-related genes, PLK4 and CENPE, were also correlate with AURKB (Fig. 4H). These findings suggest that hesperadin suppresses the growth of UM via interfering with DNA replication and mitosis.

### AURKB Upregulated the TERT Transcription by Modulating the Phosphorylation and Methylation of Histone H3

**Figure 5.**
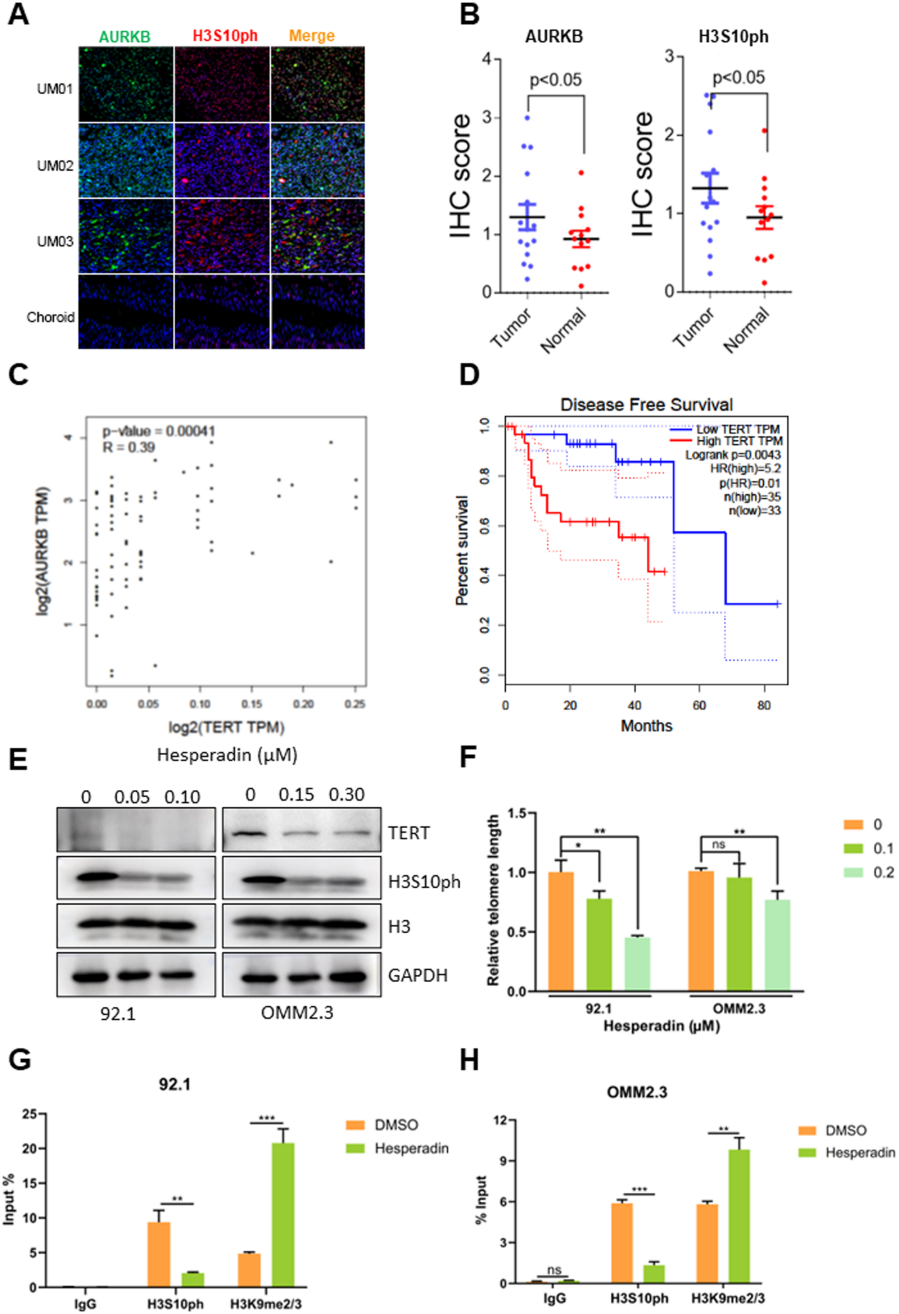
AURKB upregulated the TERT transcription by modulating the phosphorylation and methylation of histone H3. **(A)** IHC staining and **(B)** quantitation of H3S10ph and AURKB were performed in UM and paracancerous choroidal tissues. **(C)** GEPIA analysis revealed a positive correlation between AURKB and TERT expression. **(D)** Kaplan–Meier survival analysis based on GEPIA suggested TERT was negatively correlated with the overall survival rate for patients with UM. **(E)** Immunoblotting analysis of H3S10ph and TERT in 92.1 and OMM2.3 cells treated with increasing concentrations of hesperadin. **(F**, **G)** ChIP assays were conducted on both DMSO- and hesperadin-treated UM cells to detect the change of H3S10ph and H3K9me2/3. **(H)** Relative telomere length was detected using dual-labeled fluorescence probe-based RT-qPCR. The significant difference between the treated and the control was tested by the Student *t*-test using GraphPad Software 8.0.1.

To further investigate the role of AURKB in UM, we conducted an IHC analysis of our patients' tissues. The results showed that AURKB expression was higher in cancer specimens than in normal eyes (Figs. 5A, 5B). Telomere gene signature may be the downstream effector of AURKB as aforementioned. TERT expression was consistently downregulated by the AURKB inhibitor, as evident by our RNA sequencing (Fig. 4B).

To verify that TERT was the downstream effector of AURKB, we treated 92.1 and OMM2.3 with hesperadin, TERT protein was decreased dramatically (Fig. 5E). In addition, the expression of TERT was verified to correlate with that of AURKB in TCGA (Fig. 5C). Moreover, TERT overexpression was correlated significantly with poor disease-free survival in patients with UM (Fig. 5D). To further verify TERT's role in an AURKB-dominated oncogenic role in UM, we tested the drug sensitivity in TERT-overexpressed 92.1 and OMM2.3 cells (Supplementary Fig. S2). As expected, TERT induced chemoresistance of UM cells to hesperadin. It is well-known that TERT was critical in preserving telomeric integrity. Consistently, hesperadin effectively shortened the telomere length in a concentration-dependent manner (Fig. 5F). These results demonstrated that oncogenic AURKB functioned in keeping telomere intact through upregulating TERT expression.

The epigenetic states of histone H3 and chromatin accessibility are involved in cell fate decisions through transcription regulation.^12^ AURKB was a serine/threonine kinase that induces H3S10ph.^25^ To explore whether AURKB regulated its target genes in a histone modification-dependent manner, we first detected the H3S10ph levels in our patients with UM through IHC analysis. As expected, H3S10ph levels were upregulated in tumor specimens compared with normal controls (Figs. 5A, 5B). AURKB and H3S10ph colocalized to some extent, implying a possible spatial interaction (Fig. 5A, orange speckle in the merge panel). Further, immunoblotting analysis showed that H3S10ph was decreased significantly, accompanied by a concomitant decrease in TERT, after hesperadin treatment (Fig. 5E). H3S10ph was reported to support an open chromatin structure to enhance transcription by driving out the methylase complex that catalyzes H3K9me2/3.^26^ To test the hypothesis that AURKB transcriptionally regulated TERT by changing the histone phosphorylation and methylation status, we detected the H3S10ph and H3K9me2/3 of the TERT promoter using a ChIP assay. Hesperadin treatment significantly inhibited H3S10 phosphorylation, accompanied by accumulative di- and trimethylation of H3K9 (Figs. 5G, 5H). These results demonstrate that AURKB maintains an open chromatin frame to enhance the transcription of TERT via phosphorylating H3S10 and demethylating H3K9 at the TERT promoter region.

### Hesperadin Inhibited the Orthotopic Xenograft Growth

**Figure 6.**
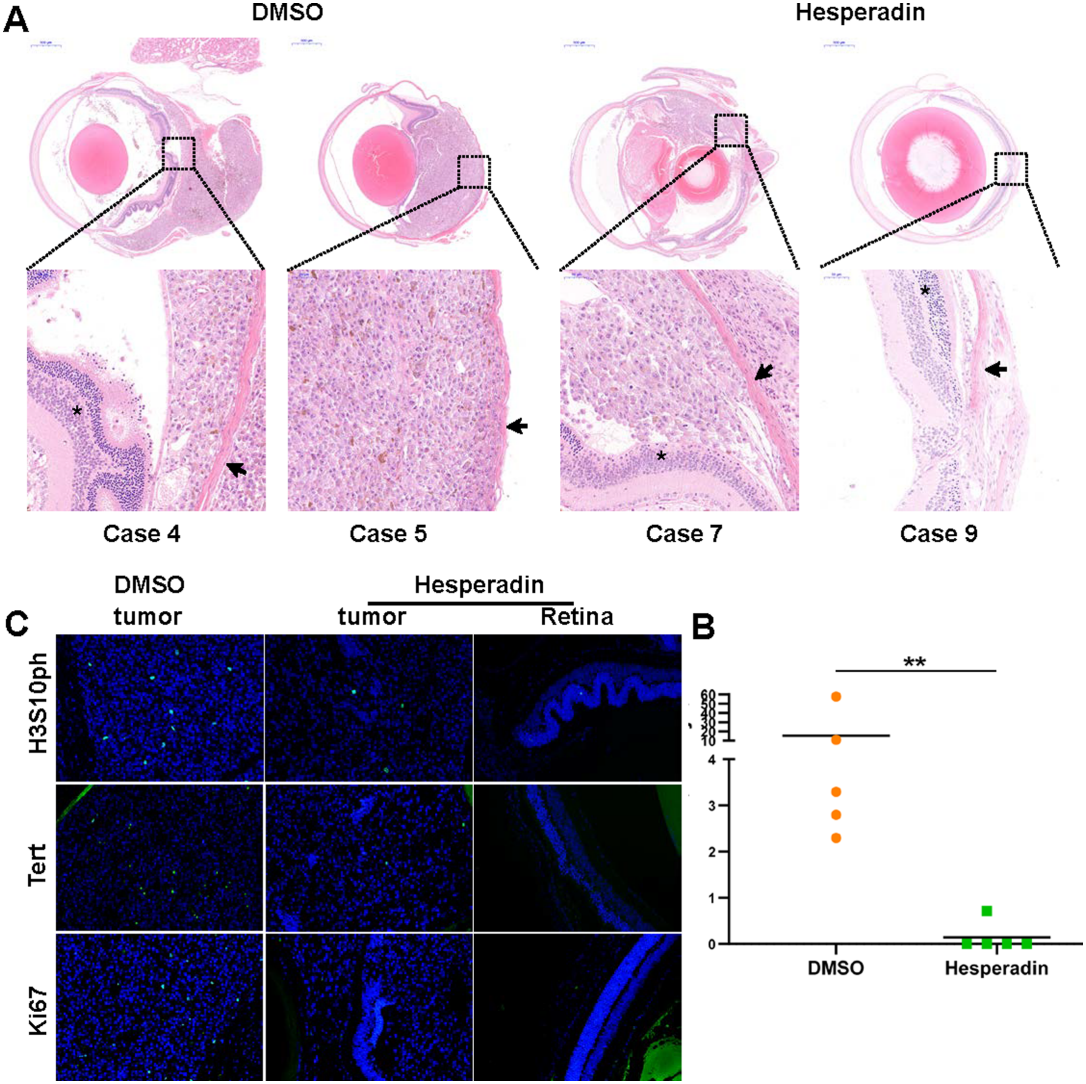
Hesperadin inhibited orthotopic xenograft growth. **(A)** Eyeballs from DMSO and hesperadin group were enucleated for HE staining. Asterisk, retina; arrowhead, sclera. **(B)** Quantitation of tumor areas at the maximal tumor section for both groups. **(C)** IHC staining of H3S10ph, TERT, and Ki67 was conducted on tumor slides from both groups. The left and middle panels showed the staining results from DMSO- and hesperadin-treated groups. The right panel presented the staining images of the eyeball section without tumors from the hesperadin-treated group. The significant difference between the treated and the control was tested by the Mann–Whitney test using GraphPad Software 8.0.1. *P < 0.05; **P < 0.01.

To evaluate the pharmacological efficiency of hespradin in vivo, we established an orthotopic xenograft model of UM. No significant weight difference was observed between the control and experimental group (data not shown), indicating the safety of hesperadin. Surprisingly, all five mice of the control group developed orthotopic tumors. However, the tumor survival rate of the experimental group was only 20% (1/5) (Figs. 6A, 6B). The results of HE staining revealed that four out of the five xenografts in the control group broke through the sclera to form extraocular tumors, whereas the only xenograft in the hespradin group was strictly restricted within the eyeball (Fig. 6A). This preclinical trial demonstrated the excellent antitumor efficiency of hesperadin in UM. To further verify the effector pathway of hesperadin in UM, we conducted IHC staining of H3S10ph, TERT, and Ki67 (Fig. 6C). The Ki67 index was decreased drastically by hesperadin treatment, confirming the antiproliferation role of hespradin. As expected, hesperadin in vivo significantly prevented the H3S10ph deposition and consequently impeded the transcription of TERT. The animal experiments confirmed that hesperadin could inhibit UM occurrence and development significantly.

## Discussion

The amplification or overexpression of AURKB has been reported in leukemia and most solid tumors.^27^^–^^31^ Moreover, AURKB expression has potentiated drug resistance in glioblastoma and lung cancer.^28^^,^^32^^,^^33^ More interestingly, AURKB played a driver role in facilitating the transformation of murine epithelia into the mammary tumor by inducing polyploid and consequent genomic instability.^34^ However, AURKB's role in UM remains elusive. A Kaplan–Meier survival analysis based on TCGA showed that higher expression of AURKB was associated with a lower overall and disease-free survival probability, indicating a poor prognosis for patients with UM. Further IHC staining of our own patients' samples demonstrated that AURKB was overexpressed aberrantly in patients with UM. These findings indicated that AURKB could be a therapeutic target for UM.

AURKB depletion has proven beneficial in decreasing tumor progression and resensitizing cancer cells to chemotherapy and target therapy.^10^^,^^18^^,^^28^ Thus, various small molecule inhibitors targeting AURKB have been developed.^11^ Highly selective inhibitors for AURKB, including hesperadin and barasertib, significantly inhibited human breast, prostate, lung, colon, and blood cancer cell proliferation in vitro and in vivo.^14^^,^^35^ Apart from preclinical studies, these inhibitors also achieved therapeutic benefits in certain solid tumors and leukemia in different phases of clinical trials.^11^ Drug sensitivity assay demonstrated that UM cell lines were prominently sensitive to the AURKB-specific inhibitor, hesperadin, as well as the pan-Aurora kinase inhibitors, danusertib and TAK-901. Nevertheless, Aurora A inhibitor I, highly selective for AURKA, exhibited much weaker antitumor activity than AURKB-targeting inhibitors, suggesting that AURKB, rather than AURKA, led to the tumorigenesis of UM. Genetic knockdown assay further verified AURKB's oncogenic role in UM development. Consistent with in vitro results, hesperadin impeded the propagation of cancer cells in eyeballs significantly. Intriguingly, hesperadin played an unexpected role in restricting UM invasion, suggesting its potential role in metastasis prevention. Further metastasis-related in vitro and in vivo experiments are needed to test this possibility.

As one of the classical mitotic drivers, AURKB ensures the faithful distribution of chromosomes into daughter cells. AURKB's abnormality led to aberrant cell division and aneuploidy/polyploidy, subsequently resulting in tumorigenic transformation.^9^^,^^36^ Wang et al.^37^ described an antagonistic interplay between AURKB and BRCA1/2 wherein they encouraged tumor progression through regulating cell cycle, chromosome tetraploidy, and cytokinesis in a p53-dependent manner. In line with previous studies, hesperadin treatment triggered striking G2/M cell cycle arrest and apoptosis in UM cells. Another evolutionarily conserved role for AURKB was to remove the torsion at replication forks and restart DNA duplication during interphase, which was triggered by the ATM/ATR-CHK1/2 pathway in response to DNA damage.^7^ Our RNA sequencing analysis showed that hesperadin impaired the genome's self-duplication significantly and halted the cell cycle progression of UM cells. Furthermore, TOP2A and MCM6, known for their roles in releasing torsion and activating replication fork,^7^^,^^38^ were transcriptionally inhibited by hesperadin treatment. Hesperadin also decreased the expression of PLK4 and CENPE remarkably, ensuring normal cell cycle transition by regulating centriole duplication and chromosome segregation.^38^ Notably, the expression of these essential genes was well-correlated with that of AURKB in patients with UM. These findings confirmed that AURKB inhibition inhibited the tumorigenesis of UM by interfering with chromatin duplication and cell cycle processes.

Intriguingly, Kyoto Encyclopedia of Genes and Genomes functional pathway analysis of TCGA revealed that telomere gene sets, widely accepted for their roles in cancer initiation and metastasis regulation, very likely participated in oncogenesis and the progression of UM under the control of AURKB. AURKB was found localized at chromosome ends and played a two-edged sword role in telomere structural integrity by interacting with protective or destructive proteins.^26^^,^^39^ It is well-known that TERT catalyzes the addition of repetitive TTAGGG sequences to the ends of chromosome to maintain genomic stability. TERT promoter mutations, which cause an increased TERT expression, are detectable in 32% to 43% of primary conjunctival melanomas and 30% of primary cutaneous melanomas, correlating with metastatic diseases and a shorter survival.^40^ However, TERT promoter mutations occur at extremely low frequency in UM. Limited literature reported that TERT was highly expressed in UM and contributed to UM stemness.^41^^,^^42^ In addition, TERT could regulate the tumor microenvironment by facilitating tumor angiogenesis, shaping the inflammation and immunosuppressive environment, as well as activating the cancer-associated fibroblasts.^43^^,^^44^ In our study, hesperadin treatment downregulated the TERT protein level and shortened the telomere length of UM cells, proving telomeric events as the effectors of AURKB.

**Figure 7.**
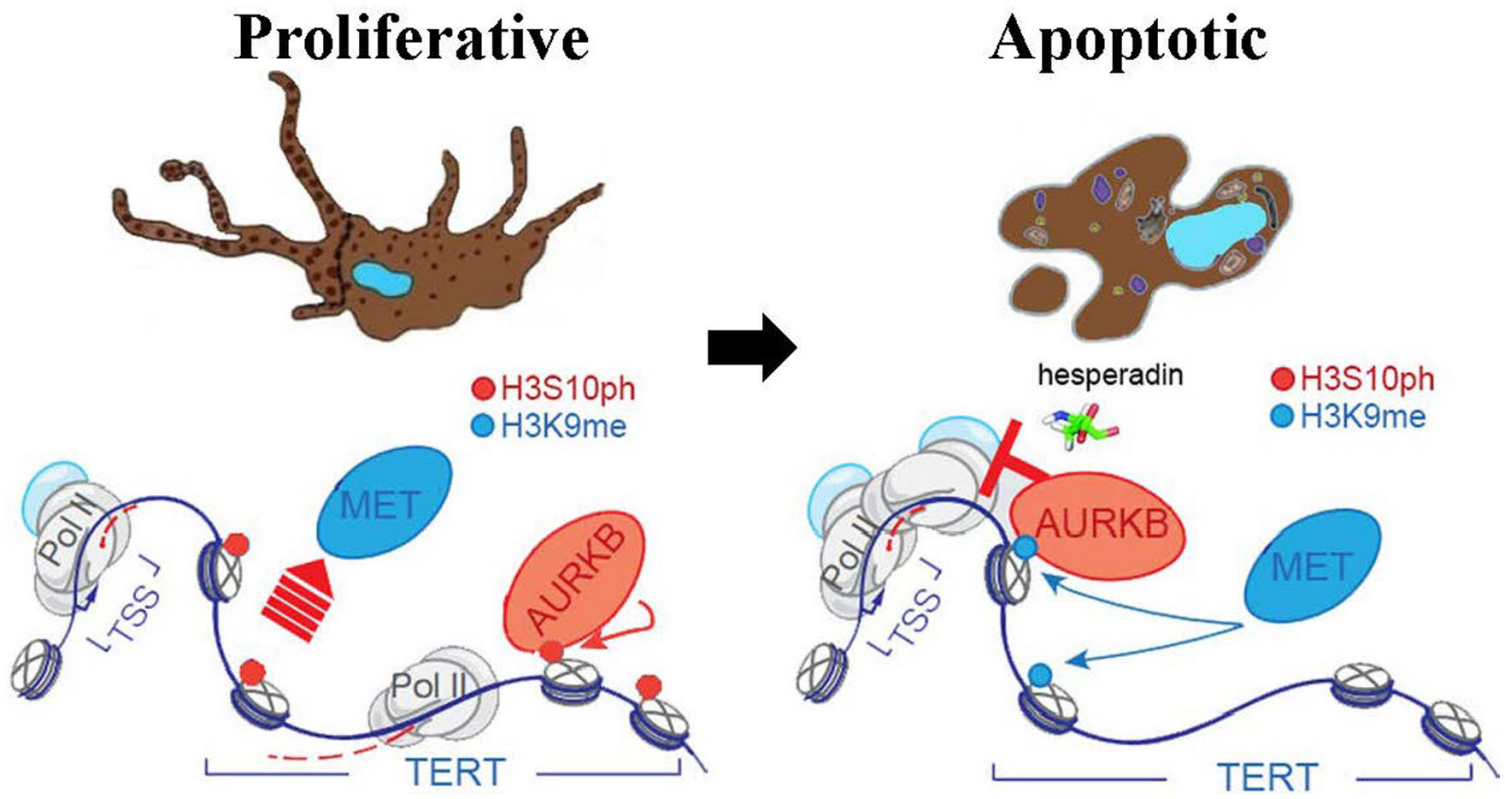
A schematic diagram of this study showing the mechanism underlying hespradin's suppressive effect on UM. AURKB expels histone methyltransferase (MET), and then phosphorylates the histone H3 at serine 10 at the promoter of TERT. Phosphorylated promoter recruits RNA polymerase complex to promote the transcription of TERT. Hespradin inhibited the enzymatic activity of AURKB, giving chance to histone methyltransferase to methylate the histone H3 at lysine 9. Methylated gene body closed the accessibility to RNA polymerase complex and inhibited the transcription of TERT, leading to apoptosis of UM.

The mechanisms underlying AURKB's regulation of molecular function were sophisticated, at least involving protein stability and chromatin remodeling. AURKB was reported to modulate chromatin accessibility by directly phosphorylating histone H3 at serine 10 in a cell cycle-dependent manner.^45^ Phosphorylated histone further expelled methylase complex aggregation and prevented consequent transrepressive H3K9me2/3 deposition.^12^^,^^46^ IHC analysis of the tissues of our patients with UM showed colocalization of AURKB and H3S10ph, indicating the possible cross-talk. Further ChIP assay demonstrated that hesperadin substantially restricted H3S10ph deposition to the promoter of TERT, giving a chance to transrepressive histone methylases. The status of H3K9me2/3 at the TERT promoter region upon hesperadin treatment condensed the chromatin and consequently closed the gate for TERT-specific transcription complex (Fig. 7). However, more research is required to identify the corresponding histone H3 kinases and methylases in UM, which may give clues to new therapeutic targets. Collectively, all these findings suggest that AURKB overexpression played an essential role in tumorigenesis of UM. Small molecule inhibitors targeting AURKB may be implicated as potential clinical antitumor candidates.

## Supplementary Material

Supplement 1
