# Supplementary material for: AURKB Enhances Chromosomal Remodeling of Telomeric Genes and Accelerates Tumorigenesis of Uveal Melanoma
Source: Invest Ophthalmol Vis Sci. 2023 Apr 20;64(4):23. doi: 10.1167/iovs.64.4.23 (PMC10132317; doi:10.1167/iovs.64.4.23)
Supplement: Supplement 1 [file iovs-64-4-23_s001.pdf]

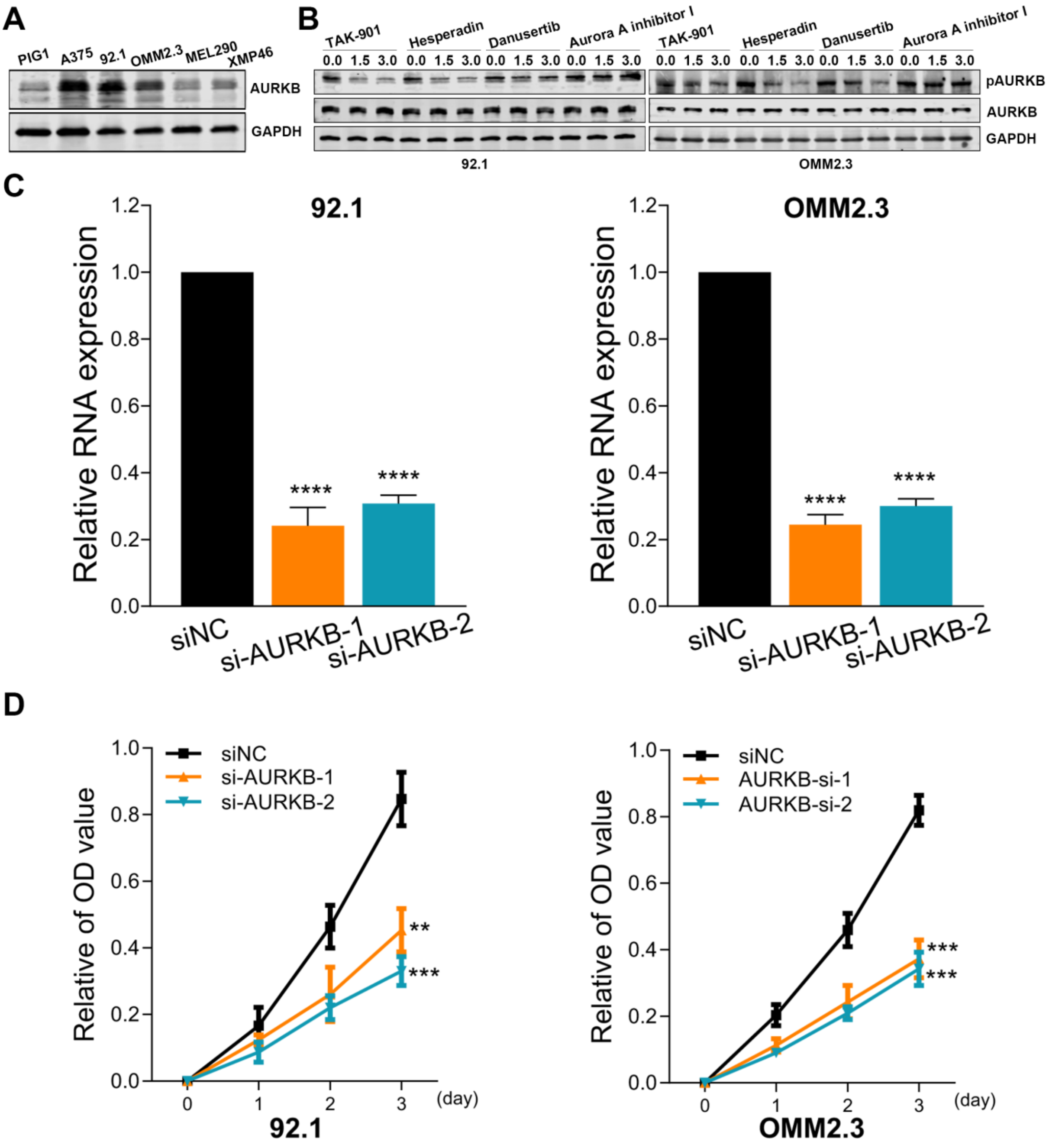

Supplementary figure 1. AURKB knockdown inhibited the growth of uveal melanoma. (A) Immunoblotting analysis of AURKB in normal control cell line PIG1 and melanoma cell lines. (B) Immunoblotting analysis of phospho-AURKB^{T232} and total AURKB in 92.1 and OMM2.3 cells treated with 0, 1.5 and 3.0 μM hesperadin for 24 hours. (C) AURKB knockdown efficiency was tested using RT-qPCR in 92.1 and OMM2.3. (D) Cell proliferation was analyzed by CCK8 assay.

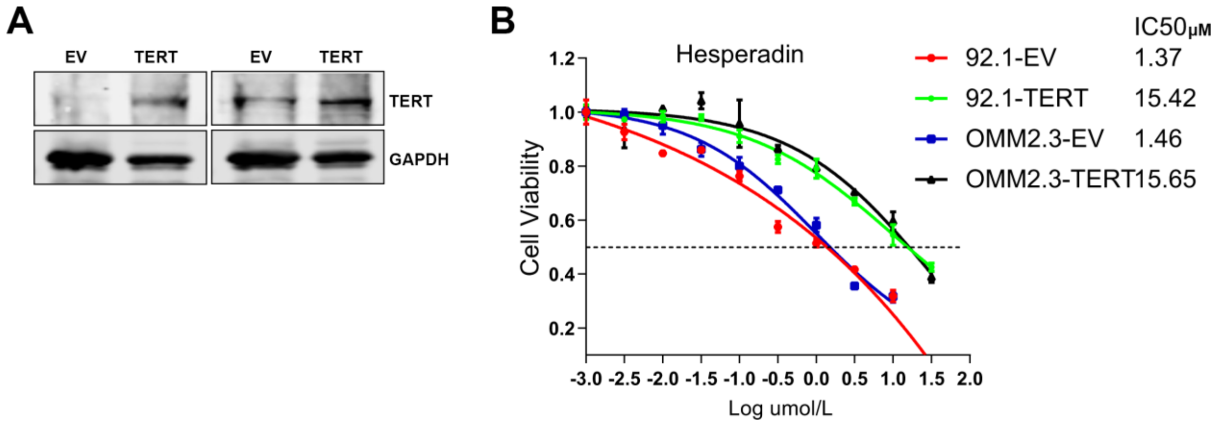

Supplementary figure 2. TERT induced chemoresistance of uveal melanoma to hesperadin. (A) Immunoblotting analysis of TERT in 92.1 and OMM2.3 with TERT overexpression. (B) Hesperadin was examined for its anti-tumor efficacy on 92.1 and OMM2.3 cells with TERT overexpression. IC50 values were calculated using GraphPad Software 8.0.1.
